# Weekly physical activity patterns of university students: Are athletes more active than non-athletes?

**DOI:** 10.1186/s40064-016-3508-3

**Published:** 2016-10-18

**Authors:** Filipe Manuel Clemente, Pantelis Theodoros Nikolaidis, Fernando Manuel Lourenço Martins, Rui Sousa Mendes

**Affiliations:** 1 Escola Superior de Desporto e Lazer, Instituto Politécnico de Viana do Castelo, Complexo Desportivo e Lazer de Melgaço – Monte de Prado, 4960-320 Melgaço, Portugal; 2 Instituto de Telecomunicações, Delegação da Covilhã, Covilhã, Portugal; 3 Department of Physical and Cultural Education, Hellenic Army Academy, Athens, Greece; 4 Polytechnic Institute of Coimbra, Coimbra College of Education, RoboCorp, ASSERT, Coimbra, Portugal

**Keywords:** Physical activity, Accelerometer, Athletes, Young adults

## Abstract

The aim of the present study was to compare weekly physical activity (PA) and obesity-related markers in athlete and non-athlete university students. One hundred and twenty-six university students (53 males, 20.46 ± 2.04 years old, and 73 females, 19.69 ± 1.32 years old) participated in this study. Participants were fitted with a tri-axial accelerometer (ActiGraph wGT3X-BT, Shalimar, FL, USA) to assess the daily PA. Anthropometric measures of height, weight, BMI and %fat mass were determined with a stadiometer and an electronic scale. The comparison indicated that male and female athletes had a significant lower percentage of body fat than did non-athletes (*p* value = 0.001; *ES* = 0.043). Athletes spent significantly more time in light PA than did non-athletes (*p* value = 0.003; *ES* = 0.024). Female athletes spent significantly less time in sedentary mode than did non-athletes (*p* value = 0.040; *ES* = 0.008). On the other hand, female athletes spent significantly more time in light PA (*p* value = 0.003; *ES* = 0.017) and vigorous PA (*p* value = 0.001; *ES* = 0.086) than did non-athletes. Despite some statistical differences with minimal effect size, the results of this study suggested proximity between PA levels of athletes and non-athletes, mainly in the case of sedentary behaviour. No significant effects were found in the variances of PA tested in this study.

## Background

The epidemic of excess body weight (overweight) and obesity has attracted scientific interest during the last decades. A population group that has been influenced by this phenomenon is university students (Cocca et al. 2014; Varela-Mato et al. 2012). This group, whose age lies between adolescence and adulthood, presents specific characteristics (e.g. emotional and physiological changes) that influence markers of excess body weight and obesity. In particular, it has been shown that changes in university students’ consumer habits and lifestyle aspects, such as physical activity (PA), might result in increased body mass (Fedewa et al. 2014; Gropper et al. 2012). Therefore, knowledge of PA levels in this population group would contribute to designing intervention programmes targeting excess body weight and obesity, through body mass control.

PA has been well-studied in university students, mainly using self-report measures (e.g. questionnaires), and it has been observed that 22–81 % (depending on nationality) of students did not meet the current guidelines for PA levels. In this context, regular participation of students in a sport activity might help increase PA levels and decrease body mass and body fat percentage (BF). However, a series of recent studies on the prevalence of excess body weight and obesity in various sport and age groups has revealed similar rates between athletes and non-athletes (Nikolaïdis 2012; Nikolaidis et al. 2015). The above-mentioned studies clearly indicated that regular participation in a sport per se does not result in low rates of body mass and BF, and identified the need for further research in markers of excess body weight and obesity in sport groups.

Nevertheless, there is a lack of information about the differences between athlete and non-athlete university students with regards to PA levels and markers of excess body weight and obesity. In addition, it is not clear whether there is a sex effect on these differences and previous studies on PA have relied mostly on subjective (e.g. questionnaire), rather than on objective, assessment methods (e.g. accelerometer) (Dinger and Behrens 2006; Peterson et al. 2015). Such information would be important for both researchers and health practitioners. Researchers focusing on PA and obesity could use such information as reference data in future studies. Moreover, the comparison between athletes and non-athletes might help health practitioners to develop optimal exercise and nutrition interventions targeting excess body weight and obesity. Therefore, the aim of the present study was to examine PA levels and markers of excess body weight and obesity in female and male athlete and non-athlete university students.

## Methods

### Participants

Participants included 126 Portuguese university students (53 male with 20.46 ± 2.04 years old and 73 female with 19.69 ± 1.32 years old). Thirty-three (26.19 %) were amateur or professional athletes with a regular practice in their clubs (3–5 weekly training sessions more one competition per week). The majority lived in rented flats or in the campus hostels with a small home-to-university distance. An informed consent form was signed for all volunteers in this study. A scientific committee from Polytechnic Institute of Coimbra, Coimbra College of Education, approved the ethical standards of this study. The study followed the ethical recommendations of Declaration of Helsinki for the study in humans.

### Procedures

Each participant was individually assessed before to wear the accelerometer. Anthropometric measures of height, weight, BMI and %fat mass were determined with a stadiometer (SAGE, precision 0.1 cm, range 0–230 cm) and an electronic scale (Tanita SC 330 S; precision 100 g, range 0–270 kg). Two measurements were carried out in the final value resulted from the average.

It was asked to participants their regular activities. It was considered a sports athlete all the participants that reported an involvement with a club, with two or more trainings plus one competition per week. Physical activity patterns without competition were not included in the sports athlete group.

After to individually assessment, participants were fitted with a tri-axial accelerometer (ActiGraph wGT3X-BT, Shalimar, FL, USA). The accelerometer was programmed to collect 10-s epochs. Participants were instructed to wear accelerometer by seven consecutive days, 24 h/day. Sleep time was also included in the time of wearing. Participants were only instructed to not use accelerometers to take shower or to water-based activities. After to finish the seventh day, participants turned to laboratory to remove the accelerometer. ActiGraph data were analysed using Actilife 6.0 software. Ten seconds epochs were collapsed into 60-s epochs that have been the protocol for the study in young adults. Sixty minutes without activity (zero counts) was considered nonwear time. This time was not included in the data treatment.

The Actilife software allowed to extract the total daily and hourly counts per minute (cpm) of sedentary time (minutes per day), light PA (minutes per day), moderate PA (minutes per day) and vigorous PA (minutes per day) and number of steps per day. The cut-off values for PA classification were: sedentary time ≤100 cpm; light PA = 100–1951 cpm; moderate PA = 1952–5724 cpm; and vigorous PA ≥ 5725.

### Statistical procedures

Gender (male and female) and relationship with sports (athlete or not) were classified as factors. The anthropometric variables (weight, height, BMI, %fat mass) and PA variables (sedentary, light PA, moderate PA, vigorous PA and number of steps) were defined as dependent variables. The two-way MANOVA was used after validating normality and homogeneity assumptions. When the MANOVA detected significant statistical differences between the two factors, we proceeded to the two-way ANOVA for each dependent variable, followed by Tukey’s HSD post hoc test (O’Donoghue 2012). Ultimately, the statistical procedures used were one-way ANOVA and Tukey HSD post hoc per factor. Effect size (ES) was presented as *η* ^2^ and interpreted using the follow criteria: no effect (*η* ^2^ < 0.04), minimum effect (0.04 < *η* ^2^ < 0.25), moderate effect (0.25 < *η* ^2^ < 0.64) and strong effect (*η* ^2^ > 0.64) (Ferguson 2009). All data sets were tested for each statistical technique and corresponding assumptions and performed using SPSS software (version 23.0, Chicago, Illinois, USA). Statistical significance was set at 5 %.

## Results

The two-way MANOVA revealed that the gender (*p* value = 0.001; *ES* = 0.751; *large effect*) and the type of practice (*p* value = 0.021; *ES* = 0.013; *no effect*) had significant main effects on the anthropometric. There was significant interaction (Pillai’s Trace = 0.044; *p* = 0.001; *ES* = 0.044; *minimum effect*) between the gender and the type of practice.

Interaction was found between factors for height (*p* value = 0.016; *ES* = 0.007; *no effect*) and %fat mass (*p* value = 0.001; *ES* = 0.012; *no effect*). No statistically interactions were found in weight (*p* value = 0.256; *ES* = 0.001; *no effect*) and BMI (*p* value = 0.661; *ES* = 0.001; *no effect*). Table 1 shows the descriptive statistics about the anthropometric characteristics of students that participated in the study.

**Table 1:** Descriptive anthropometrics characteristics of the studied participants and the comparisons between genders and type of practice

	Athletes (n = 33)	Non-athletes (n = 93)	p value	Effect size
Women (n = 73)				
Height (cm)	164.33 (6.60)*	164.77 (6.59)*	0.626	0.001
CI (95 %)	(162.70–165.97)	(164.15–165.38)		*No effect*
Effect size	0.332 *Moderate effect*	0.401 *Moderate effect*		
Weight (kg)	60.89 (7.42)*	61.41 (10.58)*	0.708	0.001
CI (95 %)	(58.35–63.43)	(60.46–62.36)		*No effect*
Effect size	0.129 *Moderate effect*	0.172 *Minimum effect*		
%fat mass	23.26 (6.24)*	24.95 (7.63)*	0.093	0.006
CI (95 %)	(21.41–25.11)	(24.26–25.64)		*No effect*
Effect size	0.527 *Moderate effect*	0.401 *Moderate effect*		
BMI	22.90 (2.91)	22.60 (3.64)	0.530	0.001
CI (95 %)	(22.02–23.78)	(22.27–22.93)		*No effect*
Effect size	0.002 *No effect*	0.001 *No effect*		
Men (n = 53)				
Height (cm)	175.79 (6.64)*	178.21 (7.89)*	0.002	0.026
CI (95 %)	(174.89–176.70)	(177.00–179.42)		*No effect*
Effect Size	0.332 *Moderate effect*	0.401 *Moderate effect*		
Weight (kg)	70.97 (11.42)*	72.49 (8.91)*	0.184	0.005
CI (95 %)	(69.62–72.32)	(70.69–74.30)		*No effect*
Effect size	0.129 *Minimum effect*	0.172 *Moderate effect*		
%fat mass	9.60 (5.00)*	11.57 (3.31)*	0.001	0.043
CI (95 %)	(9.03–10.17)	(10.81–12.33)		*Minimum effect*
Effect size	0.527 *Moderate effect*	0.401 *Moderate effect*		
BMI	22.55 (3.09)	22.61 (1.62)	0.842	0.001
CI (95 %)	(22.21–22.89)	(22.16–23.06)		*No effect*
Effect size	0.002 *No effect*	0.001 *No effect*		

* Statistically differences between men and women for a p value <0.05

**Fig. 1:**
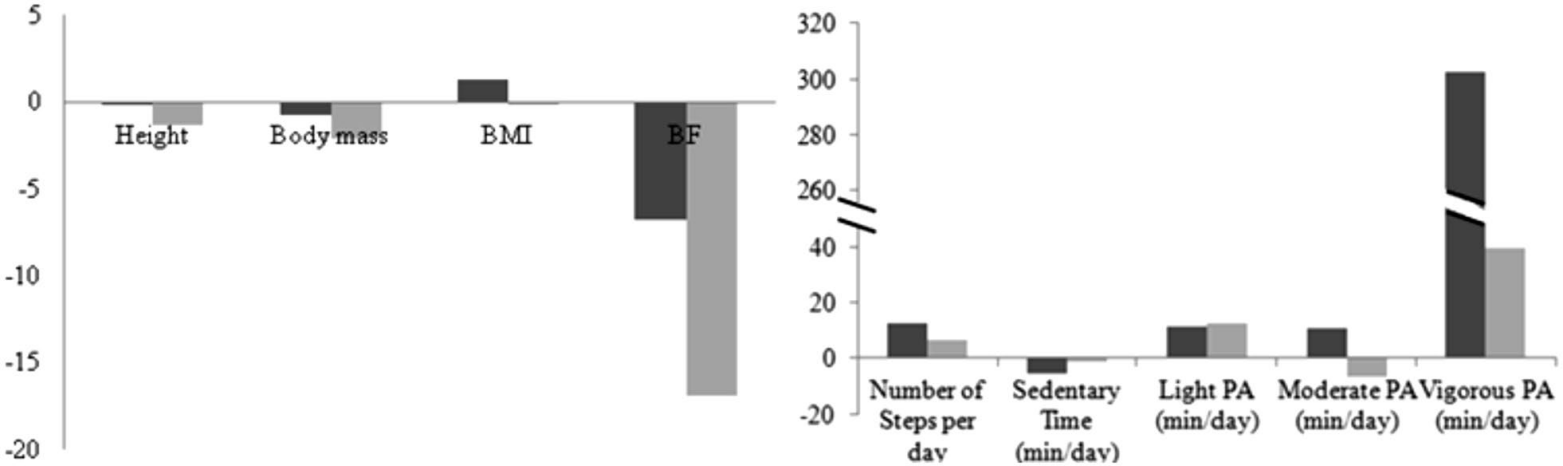
Percentage differences (%) between athletes and non-athletes in women (*dark color*) and men (*light color*) for anthropometric characteristics (*left*) and physical activity (*right*). *BMI* body mass index, *BF* body fat percentage, *PA* physical activity

The analysis of variance carried out between genders revealed that in sports athletes there were statistical differences in height (*p* value = 0.001; *ES* = 0.332), weight (*p* value = 0.001; *ES* = 0.129) and % of fat mass (*p* value = 0.001; *ES* = 0.527). It was possible to verify that male athletes are taller (6.97 %) and heavier (16.55 %). Women athletes had a greater percentage of fat mass (142.29 %). In the case of non-athletes, the analysis of variance revealed statistical differences between genders in height (*p* value = 0.001; *ES* = 0.401), weight (*p* value = 0.001; *ES* = 0.172) and % of fat mass (*p* value = 0.001; *ES* = 0.401). Similarly with athletes, the male non-athletes are taller (8.16 %) and heavier (18.04 %). Female non-athletes had a greater percentage of fat mass (115.64 %). These results can be observed in Fig. 1.

The comparison between male sports athletes and non-athletes revealed statistical differences in height (*p* value = 0.002; *ES* = 0.026) and % of fat mass (*p* value = 0.001; *ES* = 0.043). Male non-athletes were taller (1.38 %) and had a greater percentage of fat mass (20.52 %). In the case of analysis of variance carried out in female athletes and non-athletes it were found no statistical differences.

The two-way MANOVA revealed that the gender (*p* value = 0.005; *ES* = 0.019; *no effect*). No statistical differences were found on the type of practice (*p* value = 0.339; *ES* = 0.006; *no effect*). There was significant interaction (Pillai’s Trace = 0.051; *p* = 0.001; *ES* = 0.051; *minimum effect*) between the gender and the type of practice.

Interaction was found between factors for light PA (*p* value = 0.001; *ES* = 0.019; *no effect*) and vigorous PA (*p* value = 0.001; *ES* = 0.025; *no effect*). No statistically interactions were found in steps (*p* value = 0.096; *ES* = 0.003; *no effect*), time in sedentary mode (*p* value = 0.080; *ES* = 0.003; *no effect*) and moderate (*p* value = 0.880; *ES* = 0.001; *no effect*). Table 2 shows the descriptive statistics about the PA patterns of participants during one week using accelerometer.

**Table 2:** Descriptive physical activity characteristics of the studied participants and the comparisons between genders and type of practice

	Athletes (n = 33)	Non-athletes (n = 93)	p value	Effect size
Women (n = 73)				
Number of steps per day	9257.86 (4791.81)	8219.50 (38,578.58)*	0.053	0.007
CI (95 %)	(8271.74–10,243.97)	(7849.71–8589.29)		*No effect*
Effect size	0.002 *No effect*	0.019 *No effect*		
Sedentary time (min/day)	730.22 (138.22)	773.44 (158.18)	0.040	0.008
CI (95 %)	(691.64–768.81)	(758.97–787.91)		*No effect*
Effect size	0.001 *No effect*	0.005 *No effect*		
Light PA (min/day)	314.05 (75.78)	281.79 (82.05)	0.003	0.017
CI (95 %)	(293.92–334.18)	(274.24–289.34)		*No effect*
Effect size	0.001 *No effect*	0.001 *No effect*		
Moderate PA (min/day)	47.57 (41.31)	42.86 (32.35)*	0.297	0.002
CI (95 %)	(39.26–55.88)	(39.74–45.97)		*No effect*
Effect size	0.003 *No effect*	0.027 *No effect*		
Vigorous PA (min/day)	4.35 (6.72)	1.08 (2.79)*	0.001	0.086
CI (95 %)	(3.48–5.22)	(0.75–1.40)		*Minimum effect*
Effect size	0.002 *No effect*	0.071 *Minimum effect*		
Men (n = 53)				
Number of steps per day	10,228.30 (8723.13)	9597.47 (5122.31)*	0.446	0.002
CI (95 %)	(9255.40–11,201.20)	(8296.01–10,898.92)		*No effect*
Effect size	0.002 *No effect*	0.019 *No effect*		
Sedentary time (min/day)	737.94 (202.62)	746.52 (165.26)	0.677	0.001
CI (95 %)	(713.71–762.17)	(714.11–778.93)		*No effect*
Effect size	0.001 *No effect*	0.005 *No effect*		
Light PA (min/day)	311.02 (114.74)	276.65 (88.00)	0.003	0.024
CI (95 %)	(297.51–324.52)	(258.59–294.72)		*No effect*
Effect size	0.001 *No effect*	0.001 *No effect*		
Moderate PA (min/day)	52.93 (42.61)	56.66 (43.33)*	0.422	0.002
CI (95 %)	(47.46–58.39)	(49.35–63.97)		*No effect*
Effect size	0.003 *No effect*	0.027 *No effect*		
Vigorous PA (min/day)	5.17 (8.65)	3.71 (6.63)*	0.091	0.008
CI (95 %)	(4.15–6.19)	(2.35–5.07)		*No effect*
Effect size	0.002 *No effect*	0.071 *Minimum effect*		

* Statistically differences between men and women for a p value <0.05

The comparison between male and female athletes revealed no statistical differences in number of steps per day, sedentary time, light PA, moderate PA, and vigorous PA. Nevertheless, the comparison between male and female non-athletes revealed statistical differences in number of steps per day (*p* value = 0.001; *ES* = 0.019), time in moderate PA (*p* value = 0.001; *ES* = 0.027) and time in vigorous PA (*p* value = 0.001; *ES* = 0.071). Male non-athletes walked statistically more steps (16.76 %) and spent statistically more time in moderate PA (32.20 %) and vigorous PA (243.52 %). Percentage of differences in PA levels and also in anthropometric characteristics can be found in Fig. 1.

The analysis of variance between male athletes and non-athletes revealed statistical differences in light PA (*p* value = 0.003; *ES* = 0.024). Male athletes spent more time in moderate PA (12.42 %). The comparison between female athletes and non-athletes revealed statistical differences in time spent in sedentary mode (*p* value = 0.040; *ES* = 0.008), light PA (*p* value = 0.003; *ES* = 0.017) and vigorous PA (*p* value = 0.001; *ES* = 0.086). Female non-athletes spent more time in sedentary mode (5.92 %). In other hand, female athletes spent more time in light PA (11.45 %) and vigorous PA (302.78 %).

## Discussion

The aim of this study was to analyse the physical activity (PA) levels of regular athletes or non-athletes. In addition, the anthropometric characteristics were also compared. The main results revealed statistical differences in height and %fat mass between male athletes and non-athletes. Moreover, statistical differences were found in light PA of male participants and sedentary time, light PA and vigorous PA levels in female participants.

A recent study conducted in the Caucasian population in the Mediterranean area has revealed reference values of %FM between 13 and 20 % in men and 26.1 and 34.9 % for women between 20 and 29 years old (Coin et al. 2008). Our results revealed that male athletes (9.03–10.17 %FM), non-athlete men (10.81–12.33 %FM), female athletes (21.41–25.11) and non-athlete women (24.26–25.64) are below the reference values for this type of population.

The analysis of variance between male athletes and non-athletes revealed that on average, non-athletes were taller, heavier and had a greater percentage of fat mass and BMI. Nevertheless, statistical differences were only found in height and fat mass. Generally, athletes tended to have a lower percentage of fat mass and our results are in line with this idea (Katch et al. 2011; Whyte 2006). Such values can be justified by the greater recurrence of anaerobic and aerobic workouts that occur in the majority of sports, thus consuming more glycogen, carbohydrates and fat (Djelic et al. 2015). Evidence has also found that regular athletes tend to adapt their organism to an increase in energy from fat and to a decrease in energy from carbohydrates (Katch et al. 2011).

The statistical evidence found in the male participants was not confirmed in the female group. No statistical differences were found in anthropometric measures. Moreover, descriptive statistics showed that female non-athletes had a slightly larger fat mass than did athletes. Nevertheless, female athletes also had a slight greater BMI, although both were in line with healthy guidelines (Pescatello et al. 2014).

The absence of differences in fat mass between female athletes and non-athletes can be partially explained by the large amount of sedentary time spent by the athletes. In fact, the statistical differences between athletes and non-athletes in sedentary mode had no effect size, thus following a previous study that revealed that sedentary behaviour predicts some of the total and regional fatness in the female athletic population (Júdice et al. 2014).

Comparisons between male and female participants were also conducted in this study. Results revealed that male athletes and non-athletes were statistically taller, heavier and had a lower percentage of fat mass compared to female participants. These results are in line with previous studies that showed that women tend to be smaller, lighter and have a greater percentage of fat mass. The greater percentage of fat mass and the distribution of the fat by the body may be explained by the following reasons (Blaak 2001): (1) the catecholamine mediated leg free fatty acid release is lower in women compared to in men; (2) the free fatty acid release by the upper body subcutaneous fat depots is higher in men than in women; (3) there are some indications that basal fat oxidation is lower in females compared to males; and (4) postprandial fat storage may be higher in subcutaneous adipose tissue in women compared to in men.

The analysis of variance conducted in male participants revealed that athletes spend statistically more time in light PA than do non-athletes. No statistical differences were found in the remaining PA levels; nevertheless, a slightly greater average amount of time spent in vigorous activity and number of steps walked per day were found in athletes. On the other hand, non-athletes spent more time in sedentary mode and in moderate PA activities.

These pieces of evidence may lead to a thought that an athlete can be highly physically active but also can spend the rest of the day mostly in sedentary mode (Júdice et al. 2014). In fact, a study suggested that time spent in moderate-to-vigorous PA is unrelated with the time spent in sedentary mode (Craft et al. 2012). Moreover, a non-competitive athlete may ensure considerable levels of activity, and for that reason, the differences between both athletes and non-athletes can be mitigated. The comparison in female students revealed that athletes spent statistically more time in light PA and vigorous PA, as well as walked 1000 more steps per day on average, compared to non-athletes. On the other hand, non-athletes spent statistically more time in sedentary mode, although with no effect size. The minimum effect size values in all statistical differences may suggest that sports training may lead to an increase in time spent in activity but may not change the sedentary behaviour of both groups.

No statistical differences were found in PA levels between male and female athletes. Nevertheless, male non-athletes walked statistically more steps and spent statistically more time in moderate and vigorous PA levels than did female non-athletes. These results may suggest that sports may normalise the PA levels of athletes and non-competitive practice may lead to a gap between genders. This can be observed in previous studies in PA that suggested the existence of significant PA levels in male adults (Baptista et al. 2012; Bauman et al. 2009).

This study had some limitations that must be highlighted. The study design only considered athletes who are involved in three training sessions per week plus a weekly competition. For that reason, some participants classified as non-athletes may have engaged in regular physical activity in line with healthy guidelines. This limitation should be considered in future studies, particularly trying to organise three groups: athletes, non-athlete participants with regular physical activity practice and sedentary persons. It would also be interesting to analyse the sedentary patterns of athletes and track their physical activity patterns after the end of their athletic career.

The results obtained from this study may contribute to a better understanding of the PA reality in young adults. Generally, PA levels of both athlete and non-athlete participants are in line with healthy guidelines. Nevertheless, a gap between male and female non-athletes may suggest some specific behaviour that must be tracked over the course of years. Specific programmes aimed at non-athletes must be applied, mainly to promote the benefits of PA, as well as to engage non-athletes in regular PA activities over the week. In this field, special attention should be given to the female population, with attempts made to develop some activities that correspond to their expectations and aspirations.

## Conclusion

The main results of this study suggested that sports training closes the gap between physical activity patterns of male and female athletes. Nevertheless, without competitive practice, an increase of differences between male and female non-athletes can be verified. This may have serious implications over the years. It was also possible to verify that athletes and non-athletes have some similar sedentary and light PA patterns, and thus it would be interesting to track this idea in future studies with the special population of athletes.
